# Supplementary material for: MetaRibo-Seq measures translation in microbiomes
Source: Nat Commun. 2020 Jun 29;11:3268. doi: 10.1038/s41467-020-17081-z (PMC7324362; doi:10.1038/s41467-020-17081-z)
Supplement: Supplementary file 10 — Supplementary Data 7 [file 41467_2020_17081_MOESM10_ESM.zip › File2/Confidence_High_Taxonomy/82590_out.krona.html]

Javascript must be enabled to view this page.

members
magnitude
magnitudeUnassigned
count
unassigned
taxon
rank

82590\_out

149

149
superkingdom
2

149
1239
phylum

186801
class
149

149
186802
order

216572
family
39

genus
459786
39

species
1945593
3

SRS011302\_contig\_number\_29818SRS058070\_contig\_number\_15162SRS1055056\_contig\_number\_2601


SRS012849\_contig\_number\_contig-100\_5130.216192SRS013098\_contig\_number\_49719SRS013687\_contig\_number\_contig-100\_19186.64129SRS013965\_contig\_number\_39620SRS014979\_contig\_number\_26727SRS043841\_contig\_number\_6756SRS049773\_contig\_number\_417SRS049995\_contig\_number\_contig-100\_3711.202808SRS050925\_contig\_number\_4398SRS050941\_contig\_number\_18502SRS050998\_contig\_number\_contig-100\_3647.94690SRS051882\_contig\_number\_22555SRS052697\_contig\_number\_contig-100\_10690.192399SRS062654\_contig\_number\_contig-100\_8242.111709SRS065176\_contig\_number\_contig-100\_891.109382SRS075878\_contig\_number\_5865SRS076976\_contig\_number\_contig-100\_1267.45771SRS077294\_contig\_number\_contig-100\_35298.35298SRS077502\_contig\_number\_13387SRS077641\_contig\_number\_16553SRS078176\_contig\_number\_contig-100\_2407.136421SRS098644\_contig\_number\_43145SRS098717\_contig\_number\_23587SRS1041037\_contig\_number\_9998SRS104400\_contig\_number\_40181SRS1055076\_contig\_number\_4819SRS143342\_contig\_number\_20230SRS143991\_contig\_number\_27906SRS144183\_contig\_number\_31632SRS144362\_contig\_number\_35609SRS147139\_contig\_number\_36132SRS147271\_contig\_number\_37657SRS147614\_contig\_number\_contig-100\_1528.34993SRS149325\_contig\_number\_contig-100\_36710.36711SRS893256\_contig\_number\_contig-100\_1217.1218
35
species
876091

1

SRS098655\_contig\_number\_13195
2109687
species

88
genus
1392389

1297617
species
88

SRS011084\_contig\_number\_21726SRS011134\_contig\_number\_46180SRS014235\_contig\_number\_43545SRS014459\_contig\_number\_27999SRS014855\_contig\_number\_contig-100\_10889.10890SRS014923\_contig\_number\_34259SRS015065\_contig\_number\_17601SRS015431\_contig\_number\_contig-100\_13110.319399SRS015663\_contig\_number\_33104SRS016018\_contig\_number\_17239SRS016203\_contig\_number\_11554SRS016335\_contig\_number\_38320SRS017307\_contig\_number\_9929SRS017521\_contig\_number\_30569SRS017821\_contig\_number\_contig-100\_18691.126447SRS017916\_contig\_number\_18388SRS018351\_contig\_number\_18374SRS018427\_contig\_number\_19712SRS018836\_contig\_number\_contig-100\_3444.170127SRS018936\_contig\_number\_contig-100\_1027.48125SRS019068\_contig\_number\_87225SRS019496\_contig\_number\_contig-100\_5482.5483SRS019968\_contig\_number\_18385SRS020233\_contig\_number\_contig-100\_1897.318976SRS021484\_contig\_number\_16998SRS022609\_contig\_number\_28861SRS024331\_contig\_number\_2867SRS043701\_contig\_number\_12988SRS045826\_contig\_number\_contig-100\_18120.99610SRS046068\_contig\_number\_contig-100\_3738.3739SRS046369\_contig\_number\_16654SRS048060\_contig\_number\_13808SRS048164\_contig\_number\_contig-100\_1305.178774SRS049959\_contig\_number\_41335SRS050925\_contig\_number\_contig-100\_3620.196421SRS052697\_contig\_number\_37345SRS053356\_contig\_number\_26988SRS054590\_contig\_number\_contig-100\_879.25917SRS056273\_contig\_number\_33826SRS056519\_contig\_number\_16479SRS057478\_contig\_number\_9056SRS063040\_contig\_number\_contig-100\_4689.269679SRS063985\_contig\_number\_23678SRS064276\_contig\_number\_31424SRS074586\_contig\_number\_3176SRS075078\_contig\_number\_24309SRS075341\_contig\_number\_contig-100\_1136.159101SRS075773\_contig\_number\_36098SRS075984\_contig\_number\_22578SRS077024\_contig\_number\_15486SRS077231\_contig\_number\_26137SRS077392\_contig\_number\_30672SRS078242\_contig\_number\_4964SRS078665\_contig\_number\_7310SRS098881\_contig\_number\_contig-100\_4638.168479SRS103987\_contig\_number\_contig-100\_19178.133262SRS1041031\_contig\_number\_28353SRS1041033\_contig\_number\_29837SRS1041133\_contig\_number\_28389SRS1041140\_contig\_number\_22269SRS1041145\_contig\_number\_24170SRS1041157\_contig\_number\_contig-100\_3595.37966SRS104400\_contig\_number\_22928SRS104636\_contig\_number\_24708SRS105082\_contig\_number\_contig-100\_246.139993SRS1054691\_contig\_number\_11813SRS1055022\_contig\_number\_25463SRS1055034\_contig\_number\_8466SRS140492\_contig\_number\_34513SRS142503\_contig\_number\_60582SRS143876\_contig\_number\_31776SRS143895\_contig\_number\_48899SRS144537\_contig\_number\_47463SRS144753\_contig\_number\_8129SRS146888\_contig\_number\_11067SRS148091\_contig\_number\_contig-100\_437.97203SRS148159\_contig\_number\_57339SRS148424\_contig\_number\_35297SRS148721\_contig\_number\_contig-100\_1735.300366SRS148817\_contig\_number\_contig-100\_1851.81318SRS149075\_contig\_number\_12325SRS149784\_contig\_number\_17997SRS149879\_contig\_number\_contig-100\_266.155247SRS893253\_contig\_number\_contig-100\_4905.4906SRS893270\_contig\_number\_22395SRS893300\_contig\_number\_6098SRS893366\_contig\_number\_26737SRS893383\_contig\_number\_contig-100\_1553.205679

1

SRS142980\_contig\_number\_1890
species
172733

species
1897043

SRS011084\_contig\_number\_31989
1

species
1897048
1

SRS098571\_contig\_number\_59668

1897045
species

SRS016753\_contig\_number\_9614SRS045739\_contig\_number\_9518SRS074670\_contig\_number\_6404
3

family
541000
16

2292270
species
1

SRS017191\_contig\_number\_15505

2292180
species

SRS143085\_contig\_number\_6394SRS893231\_contig\_number\_7215
2

genus
244127
12

169435
species

SRS012273\_contig\_number\_43534SRS015217\_contig\_number\_21195SRS015694\_contig\_number\_contig-100\_330.138243SRS016056\_contig\_number\_2678SRS024388\_contig\_number\_contig-100\_20115.54781SRS047433\_contig\_number\_contig-100\_525.133460SRS055017\_contig\_number\_37403SRS065504\_contig\_number\_25352SRS077552\_contig\_number\_contig-100\_77.175479SRS142890\_contig\_number\_41524SRS147557\_contig\_number\_25527SRS893341\_contig\_number\_16332
12

1
genus
292632

1

SRS049402\_contig\_number\_9560
species
2302959
